# A282 RISK FACTORS ASSOCIATED WITH UNSUCCESSFUL HIGH-RESOLUTION MANOMETRY: FAILURE IS COMMON BUT WHAT CAN WE CHANGE

**DOI:** 10.1093/jcag/gwac036.282

**Published:** 2023-03-07

**Authors:** V Patel, D Rodrigues

**Affiliations:** Queen's University, Kingston, Canada

## Abstract

**Background:**

High-resolution manometry (HRM) is a diagnostic tool used to evaluate esophageal motor function and diagnose motility disorders. A standardized protocol is used to make an accurate diagnosis based on the Chicago Classification. Some existing literature suggests that incomplete or imperfect manometry tests are common, however; there remains a paucity of data to evaluate risk factors for failure to help clinicians determine when a study may be difficult to perform.

**Purpose:**

Our goal was to quantify how often failed tests occurred and determine specific factors that may be associated with failed HRM.

**Method:**

We retrospectively evaluated records for HRM tests performed over 1 year at our academic centre. Based on clinical experience, we identified several factors that may be associated with the success of HRM testing including the following: indications and symptoms leading to testing, patient’s age and biological sex, previous esophageal manometry history, previous esophageal/gastric surgery, previous septal repair/deviated septum, history of significant nausea/vomiting, history of anxiety/depression, history of irritable bowel syndrome, and medication use (opioids, proton pump inhibitors, calcium channel blockers, nitrates). We then compared patients with successful HRM vs. unsuccessful HRM with regard to our pre-specified risk factors.

**Result(s):**

29 HRM tests were unsuccessful from a total of 152 that were performed (19% failure rate). Reasons for failure included the inability to pass the probe through LES (55%) and the inability to tolerate the manometry probe for a minimum of 10 saline swallows (45%). After separating the failed cases from successful tests, both groups had a similar distribution of age and sex. Specific symptoms and indications did not have a significant association with unsuccessful tests. A previous history of failed manometry was associated with unsuccessful HRM (OR: 15, 95% CI 1.88 to 183.8, p=0.0156). Conversely, PPI usage was associated with fewer failed HRM tests (OR: 0.37, 95% CI 0.16 to 0.90, p=0.0343). Other medical history or medication use was not found to be associated with testing failure in our study.

**Conclusion(s):**

HRM is useful for diagnosing esophageal motility disorders, but incomplete tests are common. Although this study did not identify any factors in a patient’s medical history that could be used to predict failure in patients who have never had testing, further investigations may identify if PPI therapy can make HRM testing more tolerable. Additionally, the association between previous failed HRM and repeat failures suggests that endoscopic probe placement techniques should be considered instead of retrying conventional probe placement.

**Please acknowledge all funding agencies by checking the applicable boxes below:**

None

**Disclosure of Interest:**

None Declared

